# Efficacy of gel-based artificial saliva on *Candida* colonization and saliva properties in xerostomic post-radiotherapy head and neck cancer patients: a randomized controlled trial

**DOI:** 10.1007/s00784-020-03484-1

**Published:** 2020-08-10

**Authors:** Aroonwan Lam-ubol, Oranart Matangkasombut, Dunyaporn Trachootham, Supanat Tarapan, Vanthana Sattabanasuk, Sineepat Talungchit, Wannaporn Paemuang, Tawaree Phonyiam, Orapin Chokchaitam, On-ong Mungkung

**Affiliations:** 1 Department of Oral Surgery and Oral Medicine, Faculty of Dentistry, Srinakharinwirot University, 114 Sukhumvit 23, Wattana, Bangkok, 10110 Thailand; 2 Department of Microbiology and Research Unit on Oral Microbiology and Immunology, Faculty of Dentistry, Chulalongkorn University, Bangkok, Thailand; 3 Laboratory of Biotechnology, Chulabhorn Research Institute, Bangkok, Thailand; 4 Institute of Nutrition, Mahidol University, Nakhon Pathom, Thailand; 5 Langsuan Hospital, Chumphon, Thailand; 6 Faculty of Dentistry, Mahidol University, Bangkok, Thailand; 7 Department of Vascular Surgery, Faculty of Medicine Siriraj Hospital, Mahidol University, Bangkok, Thailand; 8 Chonburi Cancer Hospital, Chonburi, Thailand

**Keywords:** Xerostomia, Artificial saliva, Head and neck cancer, Radiotherapy, *Candida*, Buffering capacity

## Abstract

**Objective:**

To evaluate the efficacy of an edible artificial saliva gel, oral moisturizing jelly (OMJ), and a topical commercial gel (GC dry mouth gel) on *Candida* colonization and saliva properties.

**Materials and methods:**

This study was a secondary analysis of a single-blinded randomized controlled trial conducted in xerostomic post-radiotherapy head and neck cancer patients. *Candida* colonization, stimulated salivary flow rate (SSFR), saliva pH, and buffering capacity (BC) were measured at 0, 1, and 2 months after each intervention. *Candida* colonization was quantified by colony counts and species identified by *Candida* Chromagar, polymerase chain reaction, and API 20C AUX system. Statistical significance level was 0.05.

**Results:**

A total of 56 participants in OMJ (*N* = 30) and GC (*N* = 26) groups completed the study. OMJ significantly increased saliva pH (*p* = 0.042) and BC (*p* = 0.013) after 1-month use, while GC only improved saliva pH (*p* = 0.027). Both interventions tended to increase SSFR but only GC had a significant increase at 2 months (*p* = 0.015). GC and OMJ significantly decreased the number of *Candida* species at 1 and 2 months, respectively. Both groups tended to reduce *Candida* counts but not significant.

**Conclusions:**

Both OMJ and GC saliva gels could improve saliva pH and decrease the number of *Candida* species. OMJ is superior to GC in its buffering capacity, while GC may better improve salivary flow rate. Long-term and large-scale study is warranted to test the efficacy of artificial saliva in oral health improvement.

**Clinical relevance:**

OMJ and GC gel could decrease the number of *Candida* species and improve saliva properties in post-radiation xerostomic patients.

**Trial registration number:**

Clinicaltrials.gov NCT03035825. Date of registration: 25th January 2017.

## Introduction

Radiotherapy for head and neck cancer commonly causes destruction and fibrosis of salivary glands leading to hyposalivation [1, 2]. The quantity and quality of saliva in head and neck cancer patients are dramatically declined after completion of radiotherapy. Their salivary flow rate, pH, and buffering capacity are abnormally low [3, 4]. Consequently, the patients usually suffer from dry mouth symptoms (xerostomia) as well as poor oral conditions [2, 5, 6].

Among important oral health problems, such as dental caries, atrophic mucosa, altered taste sensation, and traumatic ulcer, candidiasis is one of the most common oral sequelae in post-radiotherapy head and neck cancer patients [5–7]. Even without any symptoms or clinical diagnosis of *Candida* infection, xerostomic post-radiotherapy head and neck cancer patients were reported to have increased *Candida* colonization [4, 8]. The amplified colonization and poor saliva properties together with immunocompromised host pose head and neck cancer patients at high risk of candidiasis [9, 10]. *Candida albicans* is the most common oral species detected in both healthy and xerostomic individuals [11–14]. However, xerostomic patients are also colonized by non-*albicans* species or multiple *Candida* species, leading to a more complex oral environment and treatment difficulty [7, 15, 16].

Current management strategies for hyposalivation (reduced salivary flow) include systemic and topical options. However, systemic therapies have side effects and are ineffective for patients with impaired salivary tissue. Topical treatments, including the use of artificial saliva, are therefore more commonly recommended [1, 17, 18]. Several artificial saliva products are commercially available [19]. To our knowledge, all of them are for oral lubrication and not recommended to be swallowed. Previous studies showed that various types of artificial saliva can relieve signs and symptoms of dry mouth to some extent [20–24]. However, most of those studies were not randomized and were short-term (2–4 weeks) [20–24]. Moreover, the effects of artificial saliva on *Candida* colonization are unclear.

A novel artificial saliva called oral moisturizing jelly (OMJ) was developed by the Dental Innovation Foundation under Royal Patronage, a non-profit organization in Thailand. The product used food-grade ingredients without any preservative agents. Therefore, it can be swallowed and provides lubrication from the oral cavity through the throat, similarly to natural saliva. Our previous pre-post study evaluated the efficacy of OMJ in xerostomic elderly patients with systemic diseases. The results demonstrated that continuous intake of OMJ for 1–2 months significantly reduced signs and symptoms of dry mouth, and achieved more than 80% satisfaction [25]. In addition, continuous use of OMJ for 1 month prevented the decline of pH and improved buffering capacity [25]. However, the effects of OMJ on *Candida* colonization and saliva properties of xerostomic post-radiotherapy head and neck cancer patients are unknown.

This report was a secondary analysis of a single-blinded randomized controlled trial in xerostomic post-radiotherapy head and neck cancer patients [26]. We previously published that continuous use of OMJ for a month improved signs and symptoms of dry mouth, and increased swallowing ability [26]. In this study, we aimed to analyze the effects of OMJ, in comparison with a commercially available gel-based artificial saliva (GC dry mouth gel), on salivary flow rates, saliva pH, and buffering capacity as well as *Candida* colonization in xerostomic post-radiotherapy head and neck cancer patients.

## Methods

This was a secondary analysis of a single-blinded randomized controlled trial conducted as previously described [26]. The study was performed according to the Declaration of Helsinki and ICH-GCP. The Ethical committee of Chonburi Cancer Hospital (IRB number 7/2559); Faculty of Dentistry, Srinakharinwirot University (DENTSWU-EC26/2560); and Institute of Nutrition, Mahidol University (MU-CIRB 2017/165.0811), approved the study protocol. All participants signed written informed consent prior to data collection. The study protocol was registered at Clinical trial.gov (clinicaltrials.gov number NCT03035825).

### Study population

The trial included 72 post-radiotherapy head and neck cancer patients with xerostomic problems (*N* = 37 for OMJ and 35 for GC (control) groups) [26]. However, in this study, we analyzed data from a subpopulation of 56 who could provide saliva samples at all time points. The participants were recruited from three sites including Chonburi Cancer Hospital; Faculty of Dentistry, Srinakharinwirot University; and Institute of Nutrition, Mahidol University, Thailand. Inclusion criteria included patients who had a history of head and neck cancer, are 30–70 years old, had finished radiotherapy for at least 1 month and/or chemotherapy for at least 2 weeks, are able to use/consume the interventions without choking, can communicate in Thai, and had subjective dry mouth score ≥ 3 according to the questionnaire used in our previous study [25, 26]. Exclusion criteria were patients with mucositis grade ≥ 1, clinically diagnosed candidiasis, or systemic diseases associated with hyposalivation, such as Sjögren’s syndrome, or those taking drugs with anti-cholinergic effects such as pilocarpine and anti-depressants. Moreover, participants with normal saliva pH and high buffering capacity at baseline were excluded from the analysis. In addition, participants who did not come for both follow-up visits or developed cancer recurrence or allergy to the interventions were discontinued from the study.

### Study design, interventions, blinding, and randomization

The protocol of the trial was described in Nuchit et al. [26]. Briefly, the participants were randomly allocated to OMJ or commercially available GC dry mouth gel using a minimization method to match age, sex, and baseline subjective dry mouth score between groups. OMJ is manufactured by Dental Innovation Foundation under Royal Patronage, Thailand. While GC dry mouth gel is a product of GC Corp., Japan, groups. All examiners involved in data collection and statistical analysis were blinded. Participants were instructed to take 1–2 teaspoons of OMJ, hold in the mouth for a few seconds, and swallow. Participants in the GC group were instructed to apply approximately 500 mg (pea-sized drop) of GC dry mouth gel as a thin layer throughout the oral cavity. Both groups were asked to use the products 6 times per day or every 3 h for 2 months.

### Sample size and power calculation

The sample size of this study was calculated as described [26]. To ensure that this secondary data analysis had adequate power, post hoc power analyses for saliva properties and *Candida* colonization were performed using G power. A power of 0.87, 0.9, and 0.92 was obtained for the data of saliva pH, buffering capacity, and the number of *Candida* species, respectively.

### Data collection

The outcome measures, including salivary flow rates, saliva pH and buffering capacity, *Candida* counts, and *Candida* species, were evaluated at 0, 1, and 2 months after interventions. Demographic data, including medical history, cancer sites, detail of cancer treatment, and history of antibiotic and antifungal drug use, were retrieved from treatment records and by interviewing the participants. Subjective dry mouth scores were obtained by using a validated questionnaire as described [25, 26].

### Salivary flow rates, pH, and buffering capacity

Stimulated saliva was collected as described previously [4, 25]. Participants were asked to chew on a piece of paraffin for 1 min. Whole stimulated saliva was collected for 10 min into a sterile 50-ml tube. Stimulated salivary flow rates were calculated as milliliters per minute. Then, the pH and buffering capacity were measured by using Seven2Go pH meter S2 (Mettler Toledo, Switzerland) equipped with ultramicroelectrode. A saliva pH of less than 6.8 was considered acidic [25]. Buffering capacity was measured using Ericsson’s method as previously described with modification [27]. Briefly, 50 μl of saliva was mixed with 150 μl of 5-mM HCl. After 10-min incubation, pH of the mixture was measured by the pH meter. Final pH of ≤ 4, 4.1–5.5, and ≥ 5.6 were considered low, moderate, and high buffering capacity, respectively [27].

### Candida counts and species identification

*Candida* colonization was evaluated from oral rinse samples. Participants were asked to orally gargle with 10 ml of 0.01-M sterile phosphate-buffered saline, pH 7.2 for 5 min as described previously [4]. After collection of the oral rinse, the samples were placed on ice and transported to the laboratory for analysis within 6 h. After centrifugation at 3000 rpm for 7 min, undiluted and 1:10 diluted samples were cultured at 37 °C for 48 h on Sabouraud dextrose agar (Himedia, Mumbai, India) and CHROMagar *Candida* (Chromagar company, Paris, France), respectively. The number of colony-forming units (CFUs) and colony color and morphology were recorded. Plates exhibiting no growth were incubated for an additional 24 h to confirm the absence of *Candida* colonies.

*Candida* species were initially evaluated by color as appeared on CHROMagar *Candida* (*C. albicans*, green; *Candida tropicalis*, metallic blue; *Candida krusei*, pink, fuzzy; other species, white to mauve). The colonies were then isolated for polymerase chain reaction (PCR) to identify the following species using species-specific primers as specified in the parentheses according to previous studies [4, 14, 28, 29]: *C. albicans* (CAL5-NL4CAL), *Candida glabrata* (CGL1-NL4CGL1), *Candida parapsilosis* (*C*TA4-NL4LEL1), and *Candida dubliniensis* (CDU2-NL4CAL). The remaining species were identified by the API 20C AUX yeast identification system (bioMérieux, Marcy L’Etoile, France).

### Statistical analysis

Comparisons of baseline data between OMJ (study) and GC (control) groups were evaluated by Chi-square test or Fisher’s exact test for categorical data, and independent *t* test or Mann-Whitney *U* test for continuous data, as specified. Comparisons of numerical outcome measures at baseline and first and second follow-up visits of the same group were analyzed by repeated measure ANOVA or related samples Friedman’s test with Bonferroni correction for multiple comparisons. Comparisons of categorical outcome measures among baseline and first and second follow-up visits of the same group were analyzed by chi-square test or Fisher’s exact test. The prevalence of *Candida* species was analyzed using descriptive statistics. Correlation between logCFU and saliva properties was analyzed by Pearson correlation analysis. All analyses were performed with the IBM SPSS statistics version 22 and GraphPad Prism version 8. A *p* value of less than 0.05 was considered statistically significant. Post hoc power analysis was performed by the G Power version 3.1.9.3. The normality of data distributions was analyzed by the Shapiro-Wilk test. Parametric statistical tests were used only when the data passed normality test (*p* > 0.05).

## Results

### Characteristics of study population

**Fig. 1:**
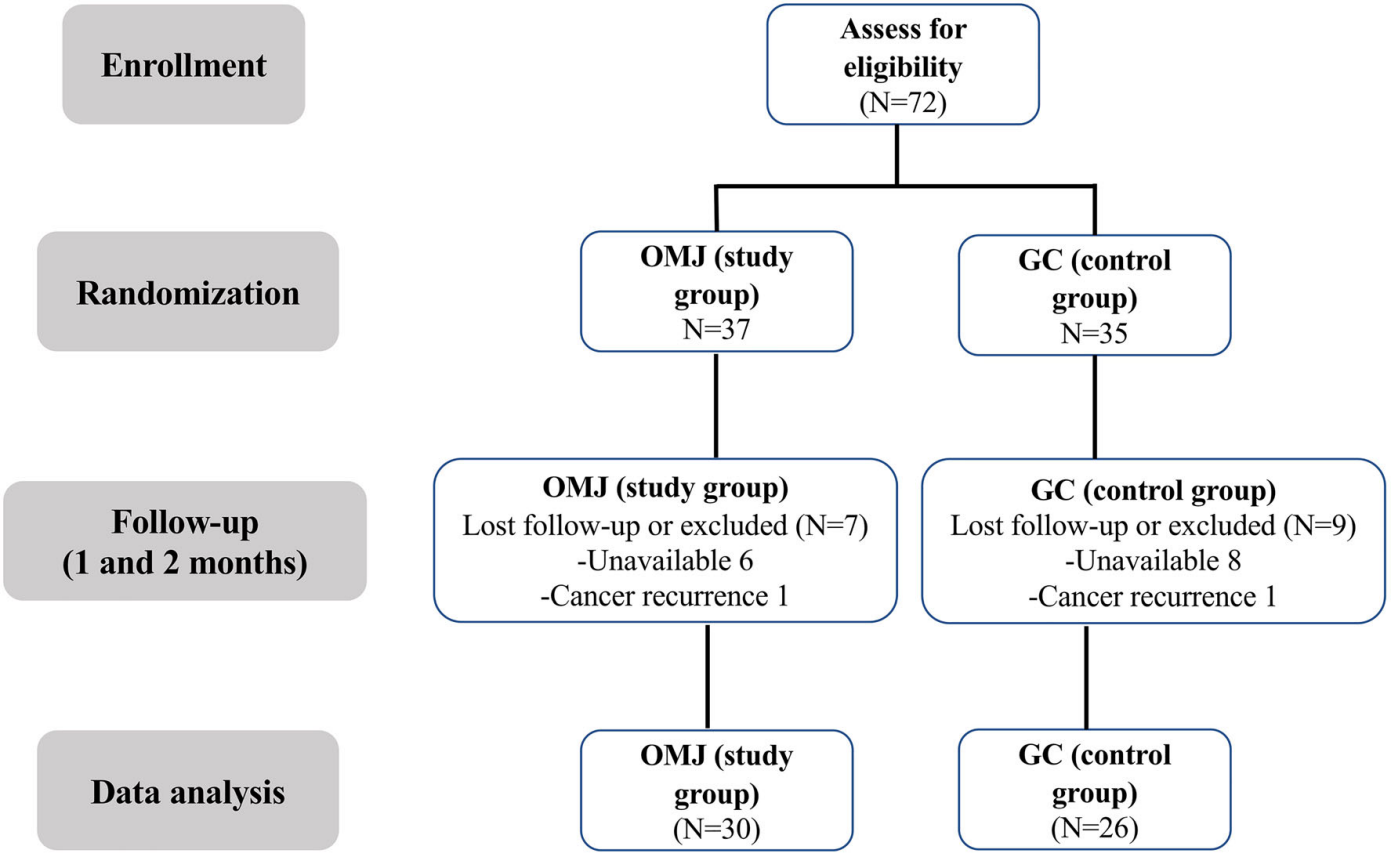
Participants’ flow chart of the randomized controlled study. Number of participants enrolled, dropped out, and included for data analysis are shown

As shown in Fig. 1, a total of 72 participants (*N* = 37 for OMJ and 35 for GC) were initially recruited and randomized in the trial as described [26]. Sixteen participants dropped out during the trial due to unavailability on appointment date and transportation difficulties (6 and 8 participants in the OMJ and GC groups, respectively) and cancer recurrence (1 participant in each group). Finally, 30 participants in the OMJ group and 26 participants in the GC group provided saliva samples at all time points and were included in this study. All participants received definitive radiotherapy for head and neck cancer, mainly by conventional (2-dimensional) technique (66%). The most common cancer sites were nasopharynx (35.7%), followed by oral cavity (28.6%) and tongue (17.9%). The majority of participants was male (67.9%) and had finished radiotherapy more than 1 year prior to enrollment (66.1%). Twelve participants (21.4%) had a history of antibiotics use within 1 month before recruitment, and 6 participants (10.7%) had a history of antifungal use within 1 year before recruitment. Twelve participants (21.4%) wore removable dental prostheses. As shown in Table 1, baseline characteristics, including dry mouth symptoms, saliva quantity and quality, and *Candida* status, of the participants in OMJ and GC groups were not statistically different (*p* > 0.05).

**Table 1:** Demographic characteristics and baseline data of the study population

	OMJ (*N* = 30)	GC (*N* = 26)	*p* value
Sex, *N* (%)			0.346^C^
Male	22 (73.3)	16 (61.5)	
Female	8 (26.7)	10 (38.5)	
Age (years, average + SD)	55.8 ± 9.3	56.9 ± 10.7	0.706^T^
Cancer location, *N* (%)			0.755^F^
Nasopharynx	10 (33.3)	10 (38.5)	
Oral cavity	10 (33.3)	6 (23.1)	
Tongue	5 (16.7)	5 (19.2)	
Larynx	3 (10)	2 (7.7)	
Oropharynx	2 (6.7)	1 (3.9)	
Others	0	2 (7.7)	
Type of radiation, *N* (%)			0.920^C^
2-dimensional	20 (66.7)	17 (65.4)	
3-dimensional	10 (33.3)	9 (34.6)	
Duration after radiotherapy, *N* (%)			0.791^F^
Less than 6 months	5 (16.7)	3 (11.5)	
6 months to 1 year	5 (16.7)	6 (23.1)	
1 year and more	20 (66.7)	17 (65.4)	
Denture use, *N* (%)	6 (20.0)	6 (23.1)	0.780^C^
Antifungal use within 1 year, *N* (%)	2 (6.7)	4 (15.4)	0.401^F^
Antibiotic use within 1 month, *N* (%)	5 (16.7)	7 (26.9)	0.351^C^
Subjective dry mouth score	5.4 ± 1.5	5.1 ± 1.5	0.520^T^
Salivary flow rates (μl/min)	80.2 ± 114.4	49.1 ± 66.5	0.274^M^
Saliva pH	6.5 ± 0.8	6.3 ± 0.9	0.322^T^
Saliva buffering capacity	4.0 ± 0.9	4.1 ± 1.1	0.808^T^
*Candida* carriers, *N* (%)	27 (90)	21 (80.8)	0.451^F^

^F^By Fisher’s exact test

^C^By Pearson chi-square test

^T^By independent *t* test

^M^By Mann-Whitney *U* test

^*^Statistically significant difference (*p* < 0.05)

### Effects of artificial saliva on salivary flow rates

**Fig. 2:**
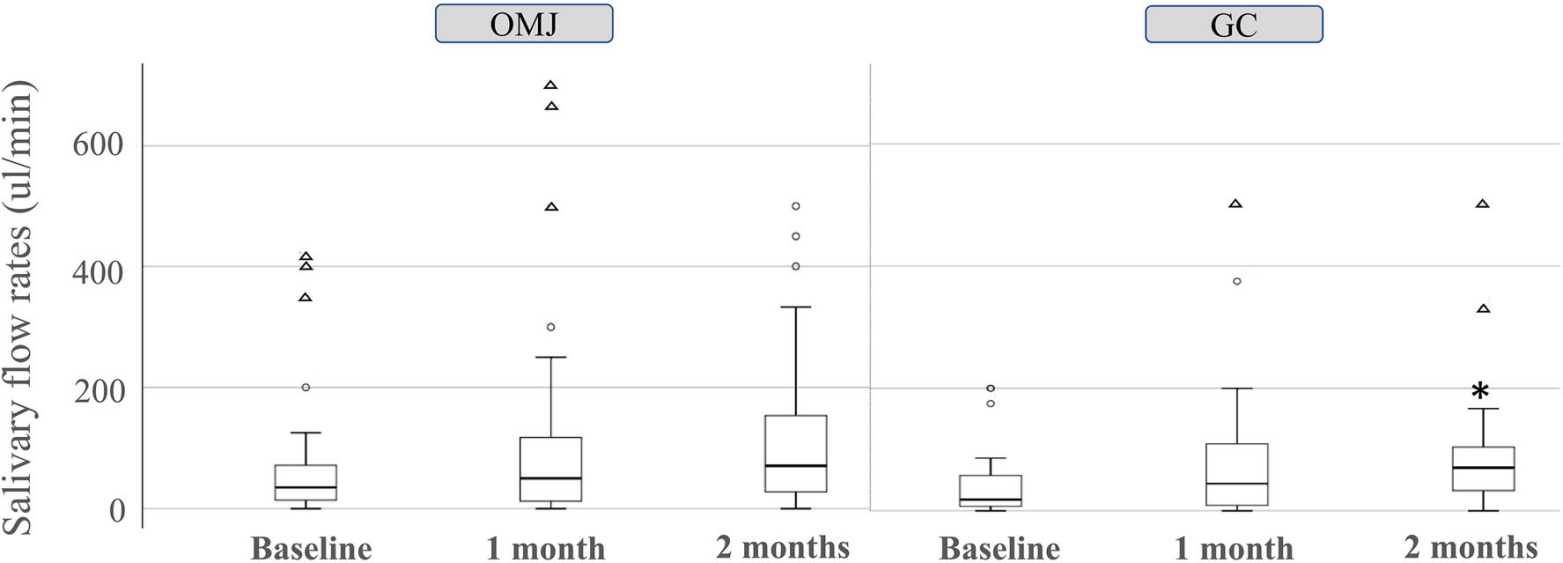
Effect of artificial saliva on stimulated salivary flow rates. Box plot represents median and interquartile (IQ) range of salivary flow rates of participants in the OMJ and GC groups at baseline and after 1 and 2 months of interventions. Whiskers indicate the highest and lowest values no greater than 1.5 times the IQ range. Open circles and triangles represent outliers (values between 1.5 and 3 times the IQ range) and extremes (values more than 3 times the IQ range), respectively. Asterisk (*) indicated a *p* value of < 0.05 by using related samples Friedman’s test with Bonferroni correction for multiple comparisons.

The stimulated salivary flow rates of participants in both OMJ and GC groups showed an increasing trend after 1 and 2 months of interventions (Fig. 2). However, only the GC group showed statistically significant improvement at 2 months (*p* = 0.015). Nevertheless, there was no statistically significant difference in the salivary flow rates between groups at each time point.

### Effects of artificial saliva on saliva pH and buffering capacity

**Fig. 3:**
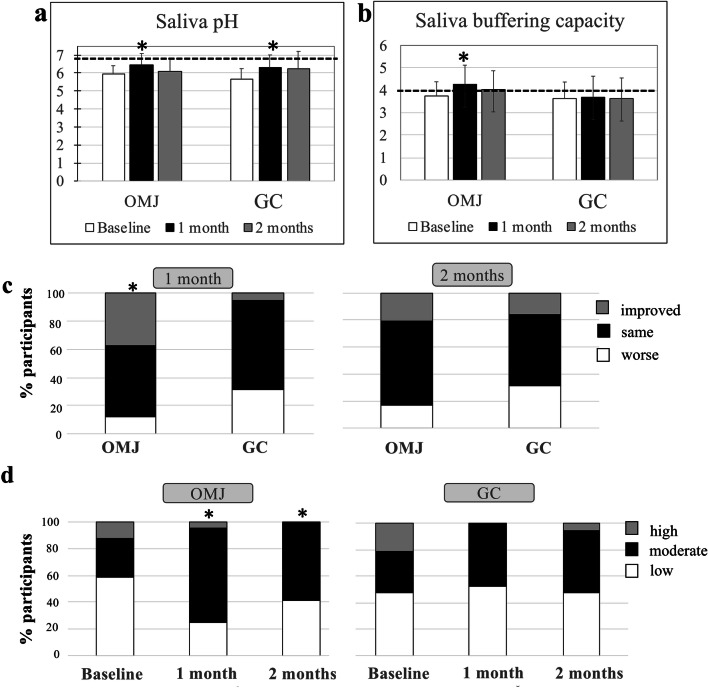
Effect of artificial saliva on saliva pH (**a**) and buffering capacity (**b**–**d**). Average saliva pH (**a**) and buffering capacity (pH after the addition of HCl) (**b**) of participants in OMJ and GC groups at baseline and after 1 and 2 months of interventions. Asterisk (*) indicates *p* value < 0.05 by using repeated measure ANOVA with Bonferroni correction for multiple comparisons. Dotted line represents the levels of neutral pH (**a**) and moderate buffering capacity (**b**). Comparison of changes in buffering capacity between OMJ and GC groups after 1 and 2 months of interventions (**c**, **d**). Stacked bars (**c**) represent percentage of participants with the same, improved, or worse buffering capacity as compared with their own baseline. Stacked bars (**d**) represent percentage of participants with low, moderate, and high buffering capacity at each time point. Asterisk (*) indicates *p* value < 0.05 by using Fisher’s exact test

As shown in Fig. 3a, participants with acidic saliva pH demonstrated a significant increase in saliva pH at 1 month after the interventions in both OMJ and GC groups (*p* = 0.042 and 0.027, respectively). However, the saliva pH did not reach neutral value (pH 6.8) and remained acidic. There was no statistically significant difference in saliva pH between groups at each time point.

Likewise, the average saliva buffering capacity of participants with abnormal buffering capacity in the OMJ group showed an improvement from low (less than 4) to moderate level at 1 and 2 months as shown in Fig. 3b. The significant difference was detected at 1 month after the use of OMJ (*p* = 0.013). On the other hand, saliva buffering capacity of those in the GC group remained low without significant change.

When analyzing changes in each subject, the OMJ group had significantly higher percentage of improved buffering capacity at 1 month than the GC group (Fig. 3c, *p* = 0.029). Moreover, the proportion of participants with moderate buffering capacity was significantly increased in the OMJ group at 1 and 2 months (Fig. 3d, *p* = 0.013 and 0.045, respectively). On the other hand, participants in the GC group did not reveal significant changes in buffering capacity.

### Effects of artificial saliva on *Candida* colonization

**Fig. 4:**
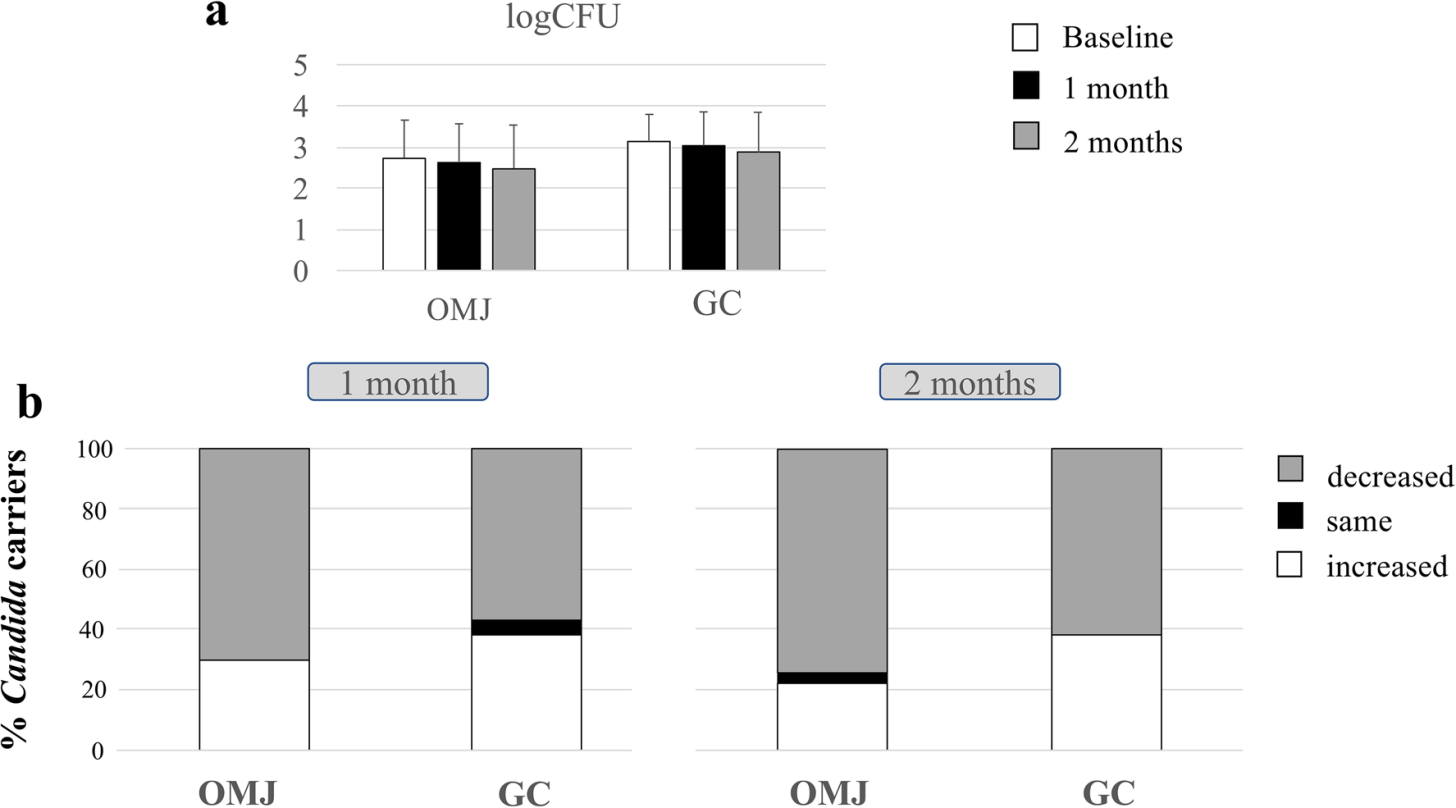
Effect of artificial saliva on the quantity of *Candida* colonization in *Candida* carriers. Average logCFU (**a**) of *Candida* colonization in OMJ and GC groups at baseline and after 1 and 2 months of interventions. Stacked bars (**b**) represent percentage of *Candida* carriers with the same, increased, or decreased logCFU as compared with their own baseline

The prevalence of *Candida* carriage in the OMJ group decreased from 90 at baseline to 80% at 2 months, while those in GC slightly decreased from 80.8 to 76.9% at 2 months. Interestingly, the quantity of *Candida* load (logCFU) among *Candida* carriers in both OMJ and GC groups slightly decreased at 1 and 2 months after using the products (Fig. 4a). However, no statistical significance was found. When we analyzed the changes in *Candida* quantity, the majority of participants in both groups showed decreased load at 1 and 2 months (70.4 and 74.1% for OMJ vs 57.1 and 61.9% for GC at 1 and 2 months, respectively) as shown in Fig. 4b. The proportion of participants with decreased *Candida* quantity than baseline in the OMJ group appeared greater than that in the GC group; however, the difference was not statistically significant. Taken together, these data suggested that continuous use of artificial saliva might decrease the amount of *Candida* colonization.

### Effects of artificial saliva on *Candida* species

**Fig. 5:**
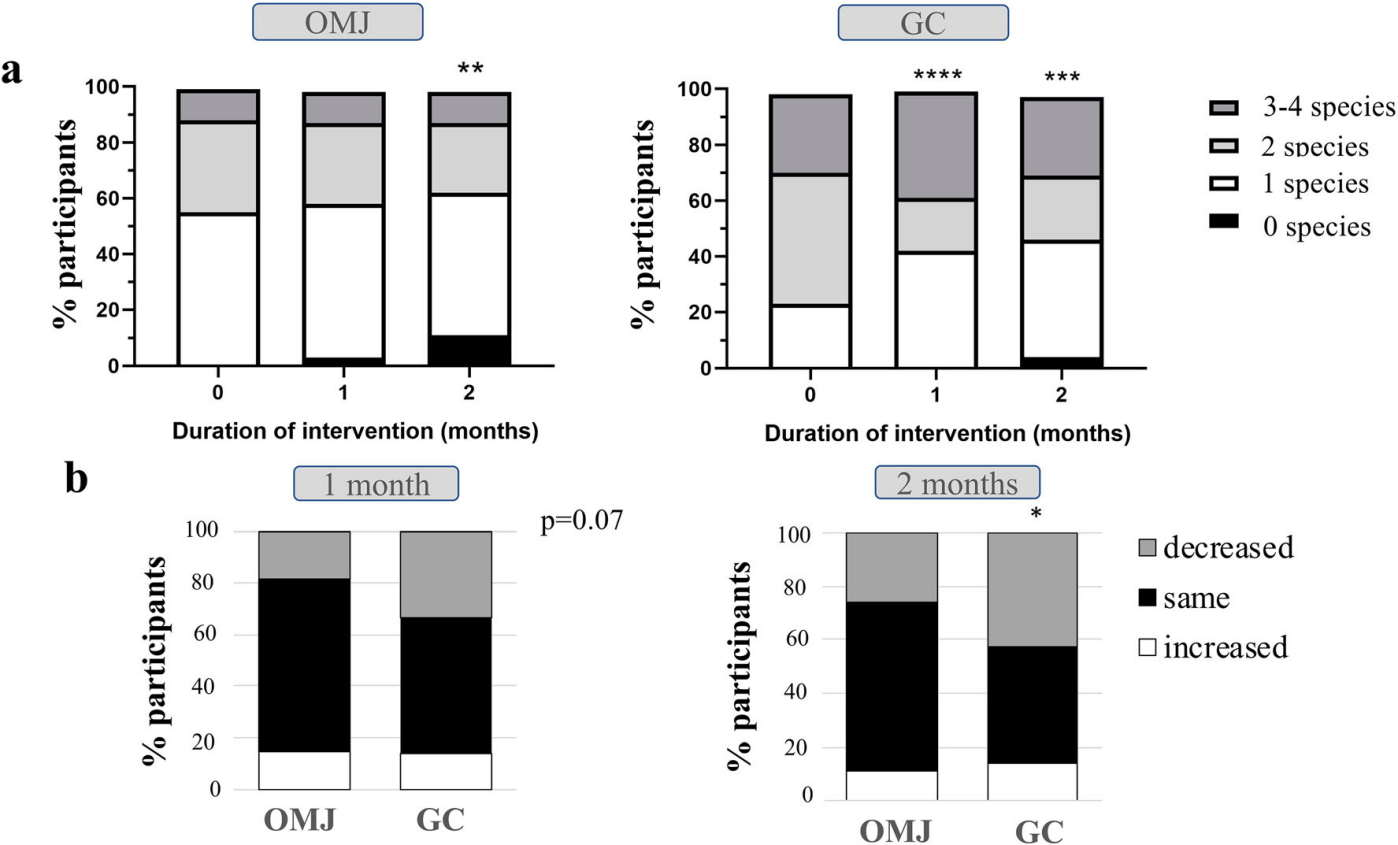
Effects of artificial saliva on the number of *Candida* species in *Candida* carriers. Stacked bar represents **a** percentage of participants with no *Candida* colonization (0 species) or colonized with 1, 2, 3, and 4 *Candida* species in OMJ and GC groups at 0, 1, and 2 months of interventions, and **b** percentage of participants with the increased, same, or decreased number of *Candida* species as compared with their own baseline in OMJ and GC groups at 1 month and 2 months of intervention. Asterisk, double asterisk, triple asterisk, and quadruple asterisk represent *p* value < 0.05, 0.01, 0.001, and 0.0001, respectively, obtained from Chi-square test

Because multiple *Candida* species were reported to affect the complexity of infection and treatment, we also identified *Candida* species colonized in the oral cavity of the participants who were *Candida* carriers. As shown in Fig. 5a, GC and OMJ significantly decreased the number of *Candida* species at 1 and 2 months (*p* < 0.0001 and *p* < 0.01), respectively. Participants in the OMJ group had a reduced number of *Candida* species from 1–2 species to no detectable *Candida* species, while the GC group had reduced mostly from 2 to 1 species.

In addition, when evaluating the changes in the number of *Candida* species in each individual, Fig. 5b shows that the GC group had significantly higher percentage of participants with a decreased number of *Candida* species at 2 months after intervention, compared with that of the OMJ group (*p* < 0.05).

**Fig. 6:**
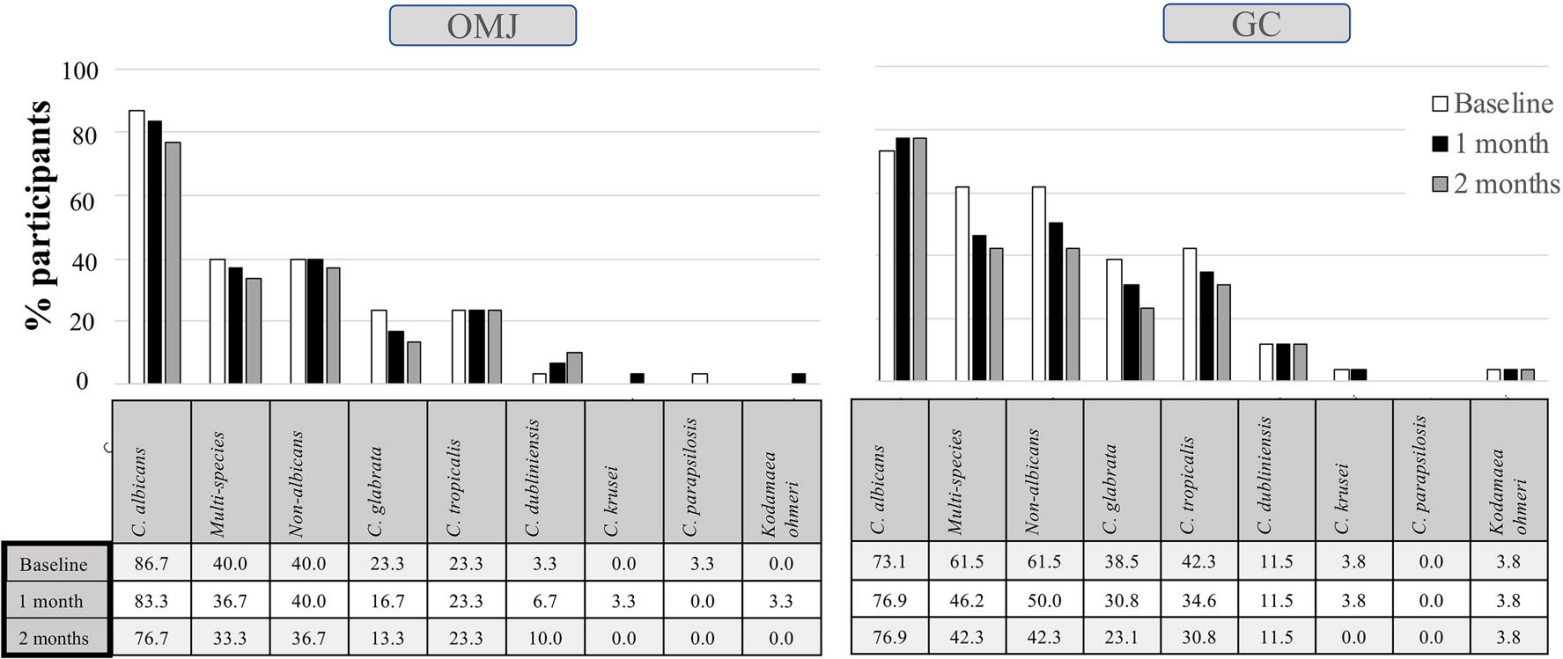
Effects of artificial saliva on *Candida* species. The prevalence of various *Candida* species detected in OMJ and GC groups at baseline and after 1 and 2 months of interventions is shown

Figure 6 shows the prevalence of all *Candida* species detected in the participants and changes during the 2-month period of the trial. The most common *Candida* species colonized in the participants was *C. albicans*, followed by *C. tropicalis*, *C. glabrata*, and *C. dubliniensis*. After 2 months, the OMJ group demonstrated a slight decrease in the prevalence of *C. albicans* (10%) and *C. glabrata* (10%), while a small increase in *C. dubliniensis* (6.67%) and no change in *C. tropicalis* were found. Participants in the GC group demonstrated a decrease in *C. tropicalis* (11.5%) and *C. glabrata* (15.4%) at 2 months, while no change in *C. dubliniensis* was observed. Interestingly, certain species, such as *C. parapsilosis* and *Kodamaea ohmeri*, were detected in both groups, but the presence may be transient.

### Correlation between saliva properties and *Candida* counts

**Fig. 7:**
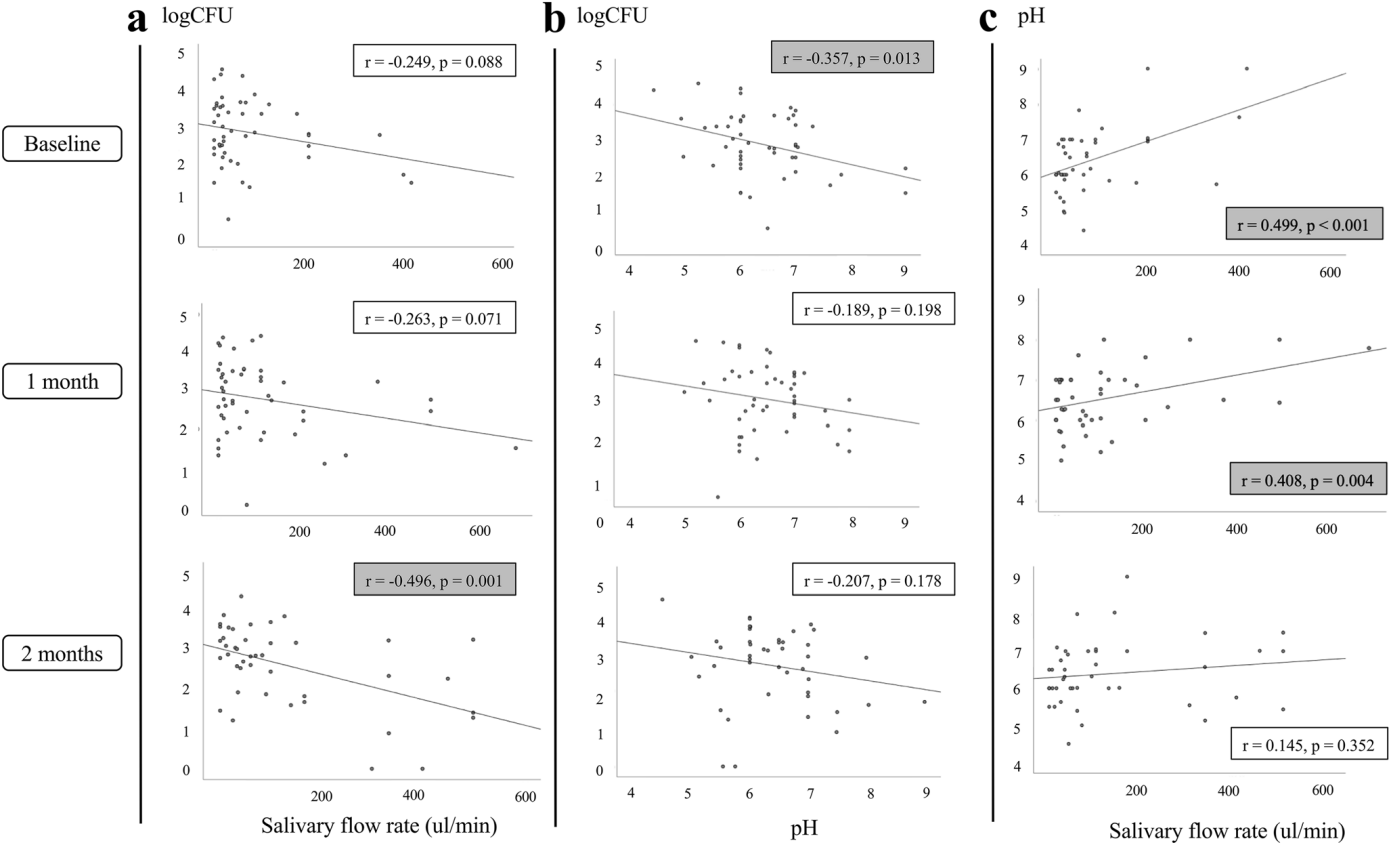
Relationship of saliva properties and quantity of *Candida* colonization after artificial saliva use. Dot plot demonstrated correlation between *Candida* logCFU and salivary flow rate (**a**), *Candida* logCFU and saliva pH (**b**), and saliva pH and salivary flow rate (**c**) of *Candida* carriers at baseline and 1 and 2 months after intervention. *r* and a *p* value analyzed by Pearson correlation analysis shown in each plot. Gray-shaded box indicated a *p* value < 0.05

To investigate the relationship between saliva properties (salivary flow rates, pH, and buffering capacity) and the quantity of *Candida* colonization (logCFU) in *Candida* carriers, correlation analysis was performed (Fig. 7). At baseline, logCFU showed significant negative correlation with saliva pH (*p* = 0.013), suggesting an increase in *Candida* colonization upon acidic environment. In addition, saliva pH had significant positive correlation with salivary flow rate at baseline and 1-month follow-up (*p* < 0.01). Interestingly, after 2-month interventions, significant negative correlation was found between *Candida* quantity (logCFU) and salivary flow rate (*p* = 0.001), but not saliva pH. No significant correlation was detected between *Candida* quantity and other saliva properties. The data suggested that the alteration of oral environment by the interventions could affect *Candida* colonization.

## Discussion

Artificial saliva is commonly used to relieve dry mouth in various groups including post-radiotherapy head and neck cancer patients [17–19]. Though hyposalivation alters saliva properties leading to complications such as candidiasis [1, 30, 31], most clinical studies of artificial saliva only focused on signs and symptoms of dry mouth [18, 20, 21, 23, 32, 33]. To our knowledge, the effect of artificial saliva on *Candida* species has never been documented. In this study, we reported for the first time that saliva gels (both OMJ and GC) could reduce the number of *Candida* species in xerostomic cancer patients. Since the higher the number of *Candida* species, the more difficult treatment can be [7, 15, 16], the effect of saliva gels suggests a favorable outcome. Furthermore, previous reports on the effect of artificial saliva on saliva biochemical properties (flow rate, pH, and buffering capacity) are inconclusive due to short-term duration and non-randomized design [34, 35]. In this study, we reported the results from a randomized controlled study with up to 2-month duration. Interestingly, both OMJ and GC improved salivary pH toward neutral one. Nevertheless, OMJ is superior to GC in its buffering capacity, while GC may better improve salivary flow rate. Interestingly, saliva pH and salivary flow rate were inversely correlated with *Candida* count. These findings suggested that modification of saliva properties may influence *Candida* colonization. Artificial saliva gel may not only alleviate dry mouth signs and symptoms but also improve saliva properties and reduce the risk of *Candida* infection.

The favorable efficacy and safety of OMJ were demonstrated in xerostomic elderly [25]. Consistently, OMJ was shown to improve signs and symptoms of dry mouth as well as swallowing ability and nutritional status in post-radiotherapy head and neck cancer patients [26]. The current study revealed the effect of OMJ in improving saliva pH and buffering capacity and reducing the number of *Candida* species. Taken all together, these evidences support the recommendation of OMJ for clinical treatment of xerostomia in elderly and cancer patients.

Due to ethical reasons, we used a commercially available product with gel-like properties, GC dry mouth gel, as a comparison group rather than a placebo. OMJ and GC dry mouth gels have similar ingredients, such as water, carboxymethylcellulose (CMC), glycerol, and flavoring agents. Although both products were CMC-based, their physical and chemical properties are different. OMJ is made from food-grade ingredients and processed by heat treatment (ultra-high temperature (UHT)). The main ingredient of OMJ is buffered water, and its viscosity is close to natural saliva. In contrast, GC dry mouth gel is more viscous and contains preservative. This is the reason why the gel is recommended for topical application to the oral mucosa and not recommended to be swallowed. On the other hand, OMJ could be held in the mouth and then swallowed to provide moisture both in the oral cavity and in the throat. Interestingly, the average saliva buffering capacity in OMJ users significantly improved at 1 month and shifted from low to moderate buffering capacity. In addition, a significantly higher proportion of participants in the OMJ group had improved buffering capacity than in the GC group. The average saliva buffering capacity of GC users remained low throughout the study. This could be because OMJ contains phosphate-buffering agent as its ingredient while GC gel does not. The improved buffering capacity was also observed in elderly patients after using OMJ [25]. In addition, a crossover study in participants with hyposalivation showed similar findings after using Biotene Dry Mouth Oral Rinse and a novel mouthwash product [36]. Because saliva buffering capacity plays an important role in maintaining a neutral oral environment, it would be of value to investigate the long-term effects of OMJ on oral and dental conditions in these patients.

The result of this study showed no significant difference between OMJ and the commercially available GC dry mouth gel in many aspects. First of all, salivary flow rates of participants in both groups appeared to improve after 1 and 2 months, although statistical significance was detected only in the GC group at 2 months. Similar finding was reported earlier in a study testing artificial saliva Optimoist in Sjogren’s syndrome and post-radiotherapy head and neck cancer patients [34] as well as another study of 1% malic acid spray in xerostomic elderly patients [37]. However, another study reported no difference [38]. While the mechanism underlying this observation is unclear, we speculate that the increased lubrication and the ability to move the tongue and oral structures may stimulate saliva flow. In addition, flavors have been reported to promote saliva production [39]. Since GC dry mouth gel has higher viscosity and stronger flavor than OMJ, it may stimulate more saliva production. We also considered the functional recovery of salivary gland tissues as a possible mechanism of increased salivary flow rates. However, such recovery has been reported to occur mostly during 6 months to 1 year after radiation, and particularly with 3-dimensional radiotherapy [2, 40]. Because the majority of our participants received 2-dimensional radiotherapy and had finished radiation for more than 1 year, the recovery during the study period would be unlikely.

Secondly, our results showed that saliva pH significantly increased in both OMJ and GC groups at 1 month, although not to a neutral value. This was in concordance with previous studies that evaluated the effects of Bioxtra gel in head and neck cancers and OMJ in elderly participants [25, 35]. In addition, Biotene Dry Mouth Oral Rinse and a novel mouthwash product were shown to improve saliva pH in xerostomic participants [36]. Both OMJ and GC products have neutral pH, which may be important for the improved saliva pH observed in the participants. This finding may be clinically important because both salivary flow rates and saliva pH were shown to correlate with reduced *Candida* colonization [41–44].

Consistently with previous studies [7, 12, 45, 46], a high prevalence of *Candida* carriage was detected in post-radiotherapy head and neck cancer patients in our study. We also found higher percentages of non-*albicans* species at baseline compared with healthy populations in other studies [4, 13, 14, 47]. After the interventions, both OMJ and GC groups had a slight decrease in *Candida* logCFU at 1 and 2 months. OMJ group appeared to have a higher percentage of users with decreased logCFU than the GC group. However, the changes/differences did not reach statistical significance. The decrease in *Candida* colonization after the use of artificial saliva had been reported by a few studies [34, 35]. However, a study that tested the effects of Biotene products, which contain antimicrobial agents, lactoperoxidase and xylitol, did not show any improvement in *Candida* count in xerostomic post-radiotherapy cancer patients [24]. This could be due to a short study time (2 weeks). The underlying mechanism of the reduction in *Candida* quantity has not been investigated. Both OMJ and GC dry mouth gel do not contain any antimicrobial agent. The possible explanation for reduced *Candida* counts in our study could be due to increased salivary flow rate and saliva pH, and, therefore, better oral clearance [41, 42]. Correlation analysis seemed to support this hypothesis. At baseline, acidic saliva pH showed significant correlation with higher *Candida* quantity and low salivary flow rate. Both interventions led to increased salivary flow rate and saliva pH (Figs. 2 and 3a). At 2-month follow-up, *Candida* logCFU showed significant negative correlation with salivary flow rate, although no significant correlation was observed with saliva pH (Fig. 7). We did not find significant correlation between *Candida* logCFU and saliva buffering capacity, but the improvement in buffering capacity in the OMJ group may also help to neutralize the acidic saliva pH. Because high amounts of *Candida* colonization were shown to be a risk factor of oral candidiasis [10], the apparent trend of decreasing *Candida* quantity after continuous use of artificial saliva suggests that it could decrease the risk of candidiasis. Further investigations with a larger number of participants and a longer follow-up time are warranted.

Similarly to previous reports, we also detected both *C. albicans* and non-*albicans Candida* species in post-radiotherapy head and neck cancer patients [4, 7, 12, 48]. Continuous usage of both OMJ and GC dry mouth gel showed a decreasing trend in the number of *Candida* species and non-*albicans* species colonized in the oral cavity, although the results were not statistically significant. This finding was noteworthy because colonization by non-*albicans* species and multiple *Candida* species was shown to be more resistant to treatment [7]. Specifically, it was reported that *C. glabrata*, *C. tropicalis*, and *K. ohmeri* can develop resistance to azole drugs [15, 16]. Since our study observed a reduced number of *Candida* species after continuous use of either GC or OMJ, application of these saliva gels may prevent *Candida* colonization and improve treatment outcome. To our knowledge, our study is the first to report changes in the prevalence of *Candida* species after using artificial saliva. A longer-term study may be required in order to see a clear effect of artificial saliva on *Candida* colonization.

The strengths of this study were the parallel-group randomized controlled design and the long duration of 2 months, which provide a high level of evidence for clinical practice. To date, only a few randomized controlled trials have been conducted to investigate the effects of artificial saliva on oral health, and most studies used a crossover design and of shorter duration [24, 37, 49]. Moreover, the dry mouth characteristics of the participants were quite similar because we only included xerostomic patients due to definitive radiotherapy, which usually leads to severe hyposalivation. We matched the demographic and certain baseline characteristics of the participants between the two groups. The other characteristics that were not matched were also comparable. Although salivary flow rates of participants in the GC group appeared lower than those in OMJ groups at baseline, all participants were in severe hyposalivation state (flow rate of less than 700 μl/min) [32], and no statistically significant difference between groups was detected.

This study carries certain limitations. First, a larger number of participants in the GC group were lost to follow-up, leaving unequal numbers of participants in both groups. Moreover, the saliva buffering capacity cannot be measured in some participants due to an insufficient amount of saliva (6 and 7 participants from OMJ and GC groups, respectively). Although we obtained adequate power for major outcome analyses, the sample size may not be enough to detect significant differences in certain variables such as *Candida* count. In addition, our 2-month follow-up period may not be long enough to see clear effects on the quantity of *Candida* colonization, yet a trend of continued improvement was observed. Thus, a larger randomized controlled study with longer follow-up period is warranted.

To conclude, this study showed that OMJ and GC usage over a period of 2 months led to an improvement of saliva properties, a reduction in the number of *Candida* species, and a decreasing trend in *Candida* counts. OMJ achieves many properties of suitable artificial saliva, including lubrication, pH neutralization, and likely increase oral clearance. There were no statistically significant differences in these outcomes when OMJ was compared with the commercially available GC dry mouth gel. Further investigations on the long-term effects of OMJ on oral and dental health will be beneficial. The information is important for future development of artificial saliva and the management of xerostomic patients.
